# Supplementary figures and images for: The effect of gene polymorphism on ticagrelor metabolism: an in vitro study of 22 CYP3A4 variants in Chinese Han population
Source: PeerJ. 2024 Sep 24;12:e18109. doi: 10.7717/peerj.18109 (PMC11430164; doi:10.7717/peerj.18109)

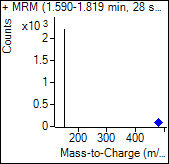

Supplement: Supplemental Information 2 [file peerj-12-18109-s002.zip › Raw figure of figure 1/bar graph of AR-124100xx.png]

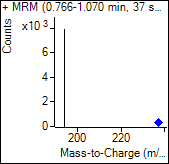

Supplement: Supplemental Information 2 [file peerj-12-18109-s002.zip › Raw figure of figure 1/bar graph of IS-MS.png]

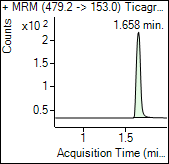

Supplement: Supplemental Information 2 [file peerj-12-18109-s002.zip › Raw figure of figure 1/chromatogram of AR-124100xx.png]

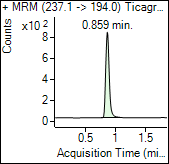

Supplement: Supplemental Information 2 [file peerj-12-18109-s002.zip › Raw figure of figure 1/chromatogram of IS.png]

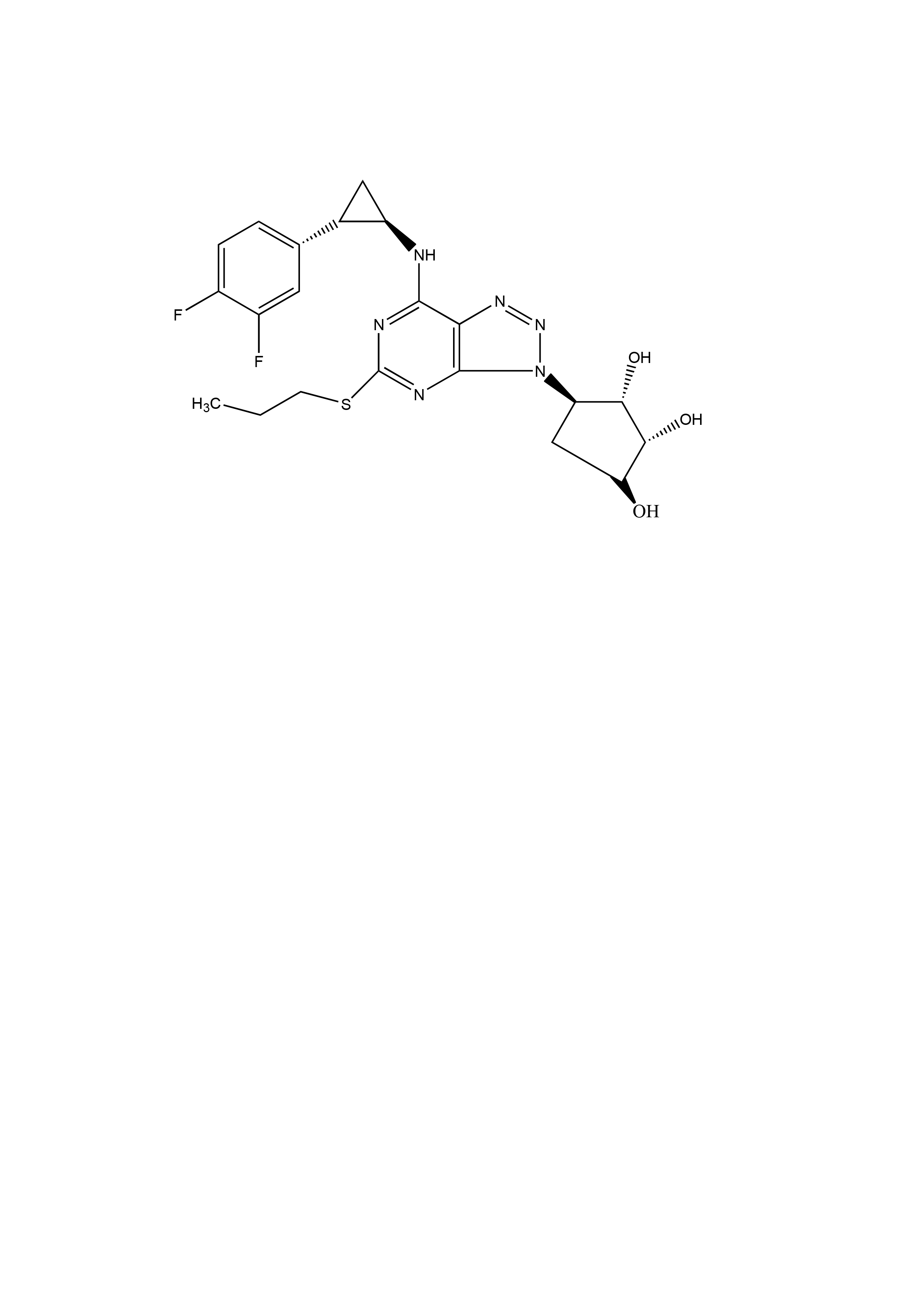

Supplement: Supplemental Information 2 [file peerj-12-18109-s002.zip › Raw figure of figure 1/structure of AR-C124910xx.tif]

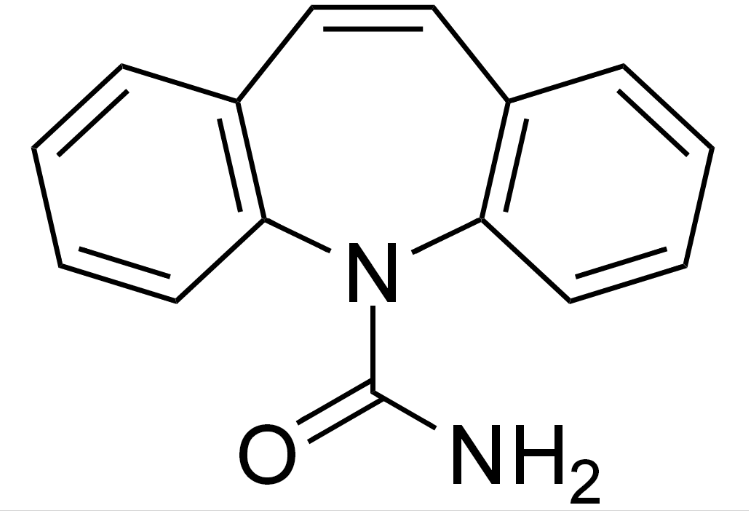

Supplement: Supplemental Information 2 [file peerj-12-18109-s002.zip › Raw figure of figure 1/structure of carbamazepine(IS).tif]
